# Neuroimaging Correlates of Post-Traumatic Stress Disorder in Traumatic Brain Injury: A Systematic Review of the Literature

**DOI:** 10.1192/j.eurpsy.2022.1198

**Published:** 2022-09-01

**Authors:** A. Esagoff, D. Stevens, M. Bray, B. Bryant, N. Daneshvari, D. Jung, C. Rodriguez, L. Richey, L. Luna, H. Sair, M. Peters

**Affiliations:** Johns Hopkins University School of Medicine, Department Of Psychiatry And Behavioral Sciences, Baltimore, United States of America

**Keywords:** Neuroimaging, Post-traumatic stress disorder, traumatic brain injury

## Abstract

**Introduction:**

Neuroimaging has been a highly utilized technique for studying traumatic brain injury (TBI) and post-traumatic stress disorder (PTSD) independently of one another, however, neuroimaging has increasingly been identified as a useful tool in better understanding TBI-related psychiatric conditions, such as PTSD.

**Objectives:**

To complete a systematic review of the literature examining neuroimaging findings in TBI-related PTSD and to highlight the current literature’s limitations in order to strengthen future research.

**Methods:**

A PRISMA compliant literature search was conducted in PubMed (MEDLINE), PsychINFO, EMBASE, and Scopus databases prior to May of 2019. The initial database query yielded 4388 unique articles, which were narrowed down based on specified inclusion criteria (e.g., clear TBI definition, clinician-diagnosed PTSD, statistically analyzed relationship between neuroimaging and PTSD, quantified time interval between TBI and neuroimaging).

**Results:**

A final cohort of 10 articles met inclusion criteria, comprising the findings of 482 participants with TBI. Key neuroanatomical findings among the included articles suggest that PTSD is associated with significant changes in whole-brain networks of resting state connectivity and disruptions in bilateral frontal and temporal white matter tracts, fronto-limbic pathways, the internal capsule, and the uncinate fasciculus (Figure 1).

**Figure 1a:**
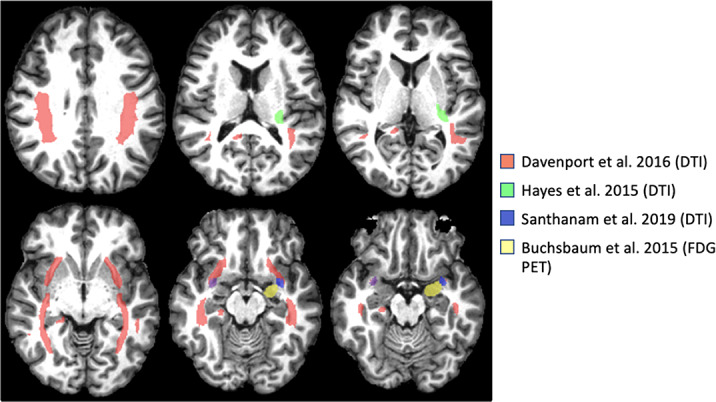
Neuroimaging Findings in TBI-related PTSD.

**Figure 1b.**
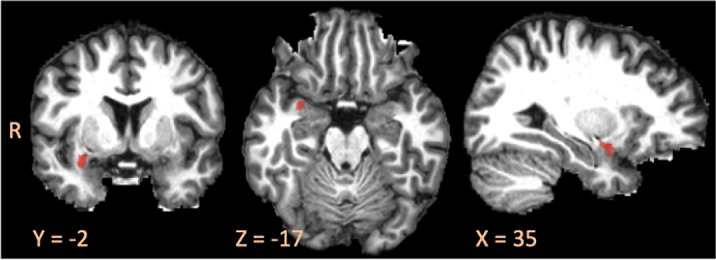
Replicated Neuroimaging Findings in TBI-related PTSD in the Right Uncinate Fasciculus.

**Conclusions:**

Additional inquiry with attention to specified imaging timing post-injury, consistent TBI definitions, clinician-diagnosed TBI and PTSD, and control groups is crucial to extrapolating discrepancies between primary and TBI-related PTSD. Prospective studies could further differentiate predisposing factors from sequelae of TBI-related PTSD.

**Disclosure:**

No significant relationships.

